# Links between type III secretion and extracytoplasmic stress responses in *Yersinia*

**DOI:** 10.3389/fcimb.2012.00125

**Published:** 2012-10-10

**Authors:** Josué Flores-Kim, Andrew J. Darwin

**Affiliations:** Department of Microbiology, New York University School of MedicineNew York, NY, USA

**Keywords:** stress response, type III secretion, Rcs, RpoE, Cpx, Psp, *Yersinia*

## Abstract

The cell envelope of pathogenic bacteria is a barrier against host environmental conditions and immunity molecules, as well as the site where many virulence factors are assembled. Extracytoplasmic stress responses (ESRs) have evolved to help maintain its integrity in conditions where it might be compromised. These ESRs also have important links to the production of envelope-associated virulence systems by the bacteria themselves. One such virulence factor is the type III secretion system (T3SS), the first example of which was provided by the pathogenic *Yersinia*. This article reviews the reported links between four different ESRs and T3SS function in *Yersinia*. Components of three of these ESRs affect the function and/or regulation of two different T3SSs. The response regulator of the Rcs ESR is involved in positive regulation of the Ysa-Ysp T3SS found in the highly pathogenic 1B biogroup of *Y. enterocolitica*. Conversely, the response regulator of the *Y. pseudotuberculosis* Cpx ESR can down-regulate production of the Ysc-Yop T3SS, and at least one other envelope virulence factor (invasin), by direct repression. Also in *Y. pseudotuberculosis*, there is some evidence suggesting that an intact RpoE ESR might be important for normal *Yersinia* outer proteins (Yop) production and secretion. Besides these regulatory links between ESRs and T3SSs, perhaps the most striking connection between T3SS function and an ESR is that between the phage shock protein (Psp) and Ysc-Yop systems of *Y. enterocolitica*. The Psp response does not affect the regulation or function of the Ysc-Yop system. Instead, Ysc-Yop T3SS production induces the Psp system, which then mitigates T3SS-induced envelope stress. Consequently, the *Y. enterocolitica* Psp system is essential when the Ysc-Yop T3SS is produced.

## Introduction

The three human pathogens in the genus *Yersinia* are *Y. pestis*, the agent of plague, along with *Y. enterocolitica* and *Y. pseudotuberculosis*, which cause food-borne gastrointestinal disease (Sukhan et al., 2001). Studies of these organisms have led to groundbreaking discoveries in the field of bacterial pathogenesis. Notably, *Yersinia* provided the first example of the multi-component structures known as type III secretion system (T3SS) that span the bacterial cell envelope to deliver virulence factors into host cells (e.g., Cornelis, 2006). In *Yersinia*, the well-studied Ysc-Yop T3SS is encoded by a *ca*. 70 kb plasmid named pYV or pCD1 that is common to the three pathogenic species (Cornelis et al., 1998). Once exported into target cells by this T3SS, the *Yersinia* outer proteins (Yop) interfere with intracellular functions that are critical for the host innate immune response (reviewed by Cornelis, 2002). The Ysc-Yop T3SS is essential for virulence in all three pathogenic species but it is not sufficient. Several other virulence determinants have also been described (reviewed by Revell and Miller, 2001). For example, during the early stages of infection, production of the invasin protein by *Y. enterocolitica* and *Y. pseudotuberculosis* promotes their transit across the intestinal epithelium. Furthermore, *Y. enterocolitica* is a heterogeneous species and strains from the highly pathogenic biogroup 1B contain virulence determinants that are absent from less pathogenic strains (Thomson et al., 2006; Wang et al., 2011). One is a chromosomal pathogenicity island (Ysa-PI) that encodes an additional T3SS known as the Ysa-Ysp system, which resembles the Mxi-Spa T3SS of *Shigella* (Haller et al., 2000; Foultier et al., 2002). There are several Ysa secreted effectors (Ysp; *Yersinia* secreted proteins) encoded throughout the chromosome (Matsumoto and Young, 2006; Witowski et al., 2008). Animal studies have indicated that the Ysa-Ysp system plays a role during initial colonization of the intestinal ileum in mice (Haller et al., 2000; Venecia and Young, 2005; Matsumoto and Young, 2006).

Relatively recent work is beginning to explore a unique aspect of T3SSs in *Yersinia*, which is the link between their production and/or function and so-called extracytoplasmic stress responses (ESRs). The Gram-negative bacterial cell envelope is the interface with the outside environment as well as being a critical structural and functional component of the cell. First, it constrains the considerable internal pressure. Second, it acts as a permeability barrier to control the movement of molecules into and out of the cell. Third, it is essential for vital cellular processes including respiration, generation, and maintenance of the proton motive force and nutrient transport. Finally, in pathogens such as *Yersinia* it is also the site where many bacterial virulence factors are assembled, including the T3SSs. Therefore, maintenance of the cell envelope is critical for survival and bacteria have evolved systems to sense and respond to potentially deleterious conditions that could damage it. These ESRs consist of signaling cascades that sense cell envelope stress and communicate with cytoplasmic regulators of gene expression to elicit a transcriptional response. The response can include the up-regulation of genes involved in mitigating the cause of the stress itself or the down-stream consequences of it. Conditions that induce ESRs include extremes of temperature, pH, and osmolarity, which might affect the cytoplasmic membrane directly or promote the misfolding and/or mislocalization of envelope proteins. In Gram-negative bacteria, the two best-characterized ESRs are the conjugative plasmid expression (CpxAR) two-component system and the RpoE/σ^*E*^ extracytoplasmic function sigma factor (ECF) system. These two systems elicit a response to the misfolding of periplasmic or outer membrane proteins (reviewed in Macritchie et al., 2008). Osmotic shock, desiccation, and overproduction of envelope proteins that are deleterious to outer membrane integrity activate another ESR known as the regulation of capsular polysaccharide synthesis (Rcs) phosphorelay system (reviewed in Huang et al., 2006). Another two-component system, the bacterial adaptive response, sensory kinase and response-regulator (BaeSR) has been described as an ESR (Raffa and Raivio, 2002). Recent findings suggest that the primary role of the BaeSR system might be to up-regulate an efflux pump in response to oxidative stress that could damage the cell envelope (Leblanc et al., 2011). Finally, the phage shock protein (Psp) response is a fifth ESR that is thought to respond to events that might compromise cytoplasmic membrane integrity (reviewed by Darwin, 2005; Joly et al., 2010; Yamaguchi and Darwin, 2012).

In addition to their importance to basic bacterial physiology, ESRs have also been linked to bacterial pathogenesis (reviewed by Hung et al., 2001; Rowley et al., 2006). First, environmental conditions in the host including elevated temperature, osmolarity, and antimicrobial peptides can affect cell envelope integrity. Second, the assembly and function of complex virulence systems has the potential to negatively affect the cell envelope directly. In fact, many studies have linked ESRs to various virulence functions of pathogenic bacteria, including the production of macromolecular apparatuses involved in motility, biofilm formation, colonization, and the secretion of cytotoxic effectors (e.g., Humphreys et al., 1999; Kovacikova and Skorupski, 2002; Wu et al., 2004; Nevesinjac and Raivio, 2005; Nishino et al., 2005; Huang et al., 2006; Macritchie et al., 2012). In *Yersinia*, regulation of the Ysc-Yop T3SS is mediated by a complex set of events that require built-in regulatory elements encoded by the pYV plasmid (Marceau, 2005) as well as some chromosomally encoded factors (e.g., Cornelis et al., 1991). In addition, it has also come to be appreciated that ESRs might impact the regulation and function of the Ysc-Yop T3SS. In particular, studies have reported regulatory and functional links between the Cpx, Psp, and Ysc-Yop systems (e.g., Darwin and Miller, 2001; Carlsson et al., 2007a). Furthermore, a regulatory link between the Rcs ESR and the chromosomally encoded Ysa-Ysp T3SS of the highly pathogenic 1B biogroup of *Y. enterocolitica* has been described (Venecia and Young, 2005; Walker and Miller, 2009). This review focuses on progress toward understanding the intrinsic and complex relationship between ESRs and T3SSs in the pathogenic *Yersinia* species.

## Extracytoplasmic stress responses that regulate T3SSs in *Yersinia*

Changes in environmental conditions encountered upon host infection such as temperature, pH and osmolarity have been associated with triggering the regulatory cascades that activate ESRs and also activating virulence factor gene expression. Therefore, placing virulence genes under the direct positive control of the transcriptional regulator component of an ESR is an efficient means to link activation of both with their common inducing signals. There is evidence for positive control of T3SS gene expression by at least one ESR regulator (RcsB) in *Yersinia*. Furthermore, T3SS production involves the assembly of numerous proteins in the cell envelope. This has the potential to compromise cell envelope integrity, especially if any of those proteins are prone to misfolding and/or mislocalization. Down-regulation of a T3SS by an ESR would be an obvious way to mitigate this stress and recent evidence suggests that the response regulator component of the Cpx ESR does this in *Y. pseudotuberculosis*. These emerging links between ESRs and the regulation of T3SS production in *Yersinia* are discussed in this section.

### The Rcs system affects expression of the genes encoding the Ysa-Ysp T3SS in highly pathogenic *Y. enterocolitica*

A link between an ESR and the regulation of genes encoding a *Yersinia* T3SS was established first for the Rcs and Ysa-Ysp systems of *Y*. *enterocolitica*. The Rcs ESR is a non-canonical two-component system found exclusively in the family Enterobacteriaceae (Huang et al., 2006). Conditions such as osmotic shock, desiccation, overproduction of envelope proteins and perturbations in extracellular polysaccharide production are inducing cues (reviewed in Huang et al., 2006). RcsC is an inner membrane sensor kinase (Stout, 1994) that becomes autophosphorylated at a conserved histidine in the presence of an inducing stimulus. The phosphoryl group is transferred to the intermediary protein RscD, also located at the inner membrane, which then transfers it to the cytoplasmic response regulator RcsB. Phosphorylated RcsB directly regulates the transcription of target genes as a homodimer, or as a heterodimer with RcsA (reviewed in Majdalani and Gottesman, 2005; Huang et al., 2006). The Rcs response has been implicated in various aspects of bacterial pathogenesis including the development of biofilms in *Escherichia coli* and Vi antigen expression in *Salmonella enterica* serovar Typhimurium (S. Typhimurium; Arricau et al., 1998; Ferrieres and Clarke, 2003). In *Yersinia*, in addition to its effects on the Ysa-Ysp system (described below), transcriptional microarray analysis has revealed a correlation between activation of the Rcs response and the expression of genes involved in adhesion, motility, biofilm formation, and resistance to bile salts (Hinchliffe et al., 2008).

**Figure 1:**
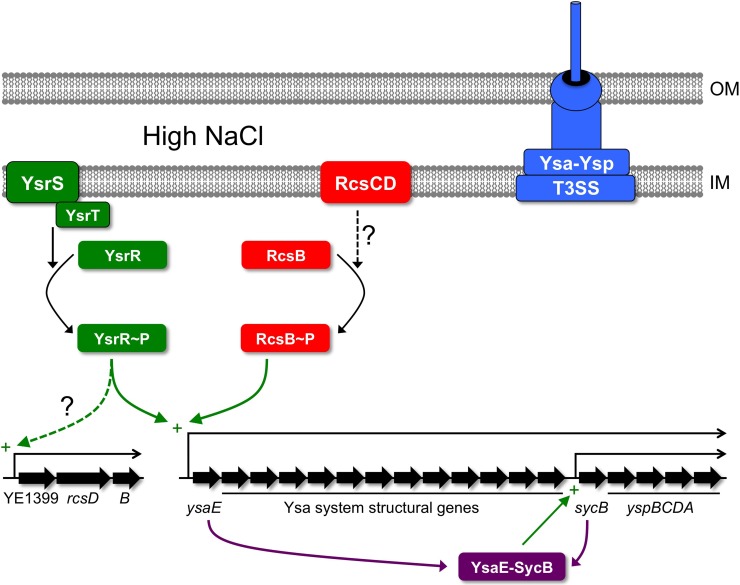
**Regulation of the Ysa-Ysp T3SS in *Y. enterocolitica*.** Increased expression of the genes encoding the Ysa-Ysp system depends on the phosphorelay system composed of YsrS, YsrT, and YsrR. High salt might be sensed by YsrS, which then phosphorylates the response regulator YsrR (with the involvement of the accessory protein YsrT). YsrR~P activates the *ysaE* promoter directly or indirectly. The result is increased levels of the proteins making up the Ysa-Ysp system, including the AraC-like regulator YsaE and the Ysp chaperone SycB. YsaE and SycB form a complex that activates a promoter up-stream of *sycB*, further elevating the levels of Ysp proteins. In addition, the YsaE-SycB complex induces other *ysp* genes located elsewhere on the chromosome (not shown). In addition to regulation by the Ysr phosphorelay, the RcsB component of the Rcs ESR also has a positive regulatory role. In this case, RcsB~P might induce the *ysaE* promoter similarly to YsrR~P, although once again it is not yet known if this regulation involves a direct interaction between RcsB~P and the *ysaE* control region. It is also not known whether the RcsCD components of the Rcs system are responsible for driving phosphorylation of RcsB during activation of the Ysa-Ysp system. Finally, the Ysr regulatory system might also play a role in activating the expression of *rcsB*. The structure of the operon containing *rcsB* is inferred from the gene arrangement in the chromosome of *Y. enterocolitica* strain 8081 (Thomson et al., 2006). OM, outer membrane; IM, inner (cytoplasmic) membrane.

Most of the genes encoding the Ysa-Ysp T3SS are located in the Ysa pathogenicity island (Ysa-PI) with the majority probably organized as a single transcriptional unit initiating from a promoter up-stream of *ysaE* and encompassing 18 genes (Figure 1; Walker and Miller, 2004). This large operon encodes regulators, structural components and exported effectors. An internal promoter located up-stream of *sycB* also drives expression of only the final five genes of this operon, a *sycByspBCDA* transcript, which encodes a chaperone and secreted effectors only (Walker and Miller, 2004). An early model proposed that expression of these genes is controlled by the YsrRS two-component system and by the AraC-like regulator YsaE/chaperone SycB pair (Figure 1; Walker and Miller, 2004). According to this model, the sensor kinase YsrS responds to elevated salt concentration by phosphorylating the response regulator YsrR. YsrR then activates expression of the large *ysaE* operon leading to accumulation of YsaE and SycB, which then work to induce the *sycB* promoter (Figure 1). Later work identified the small YsrT protein as a third component of the YsrRS system (Walker et al., 2010). Interestingly, the YsrS protein has significant similarity to the RcsC protein (Walker and Miller, 2004). This became especially noteworthy when a link between the Rcs system and expression of the Ysa-Ysp system genes was uncovered.

A screen for transposon mutants defective for Ysp secretion found an insertion in *ysrS* and also in a gene outside of the Ysa-PI that encoded an orthologue of *E. coli* RcsB (Venecia and Young, 2005). The inactivation of *ysrS* or *rcsB* reduced expression of the Ysa-PI genes, although the effect of the *rcsB* mutation was more modest (Venecia and Young, 2005). Monitoring expression of the promoterless *lacZYA* operon encoded by the transposon within *rcsB* revealed that *rcsB* expression is regulated similarly to other genes within the Ysa-PI (maximal expression in early logarithmic phase, high salt concentration and neutral to alkaline pH; Venecia and Young, 2005) and dependent on YsrS. This led to the proposal that the YsrRS phosphorelay system is the dominant up-stream regulator that controls both the genes encoding the Ysa-Ysp system and also *rcsB*. Changes in the level of RcsB might then impose additional modulatory effects on *ysa-ysp* expression.

The link between RcsB and the Ysa-Ysp system has been corroborated. In frame deletions of *rcsB*, *ysrS*, or *ysrR* lead to a noticeable decrease in transcript levels of most of the Ysa-PI genes including the regulatory genes *sycB/ysaE* (Walker and Miller, 2009). These mutations also decrease the expression of some *ysp* genes that are located outside of the Ysa-PI. However, it is unlikely that RcsB (or YsrS) is a direct regulator of these *ysp* genes. Epistasis experiments suggest that the effect of the *rcsB* null mutation on *ysp* expression is an indirect consequence of reduced *ysaE* promoter activity, which reduces the levels of YsaE and SycB (Walker and Miller, 2009). YsaE and SycB are the probable direct regulators of the *ysp* genes (Figure 1). Nevertheless, RcsB is still involved in controlling expression of Ysa-Ysp system genes, potentially by both direct (the *ysaE* promoter) and indirect (*sycB* promoter and *ysp* promoters) mechanisms. However, evidence for direct regulation of the *ysaE* promoter by RcsB has not been reported.

It is clear that RcsB positively influences expression of Ysa-Ysp T3SS genes. However, one aspect that is unclear is whether *rcsB* expression itself is co-ordinately regulated with the *ysa-ysp* genes *via* YsrRS-dependent activation. One study presented evidence that it might be (Venecia and Young, 2005) and one that it might not (Walker and Miller, 2009). Perhaps this has something to do with the different approaches used. Venecia and Young used an *rcsB-lacZYA* fusion generated by insertional mutagenesis, presumably inactivating RcsB, whereas Walker and Miller measured mRNA level with the *rcsB* gene intact. What if both RcsB and YsrS control *rcsB* expression redundantly? In this hypothetical scenario, in an *rcsB*^+^ strain the introduction of a *ysrS* mutation might not affect *rcsB* expression. Conversely, with *rcsB* already inactive in the *lacZYA* insertion mutant, the subsequent loss of YsrS would have an effect.

Regardless of the mechanistic details, positive control of the Ysa-Ysp system by RcsB suggests that this might be a case where the bacterial cell is taking advantage of an activated ESR to also induce a virulence determinant. This means that Rcs-system activating signals are presumably present in the environment where Ysp effector functions are needed. It is interesting that the YsrS sensor kinase is similar to the RcsC sensor kinase of the Rcs ESR (Walker and Miller, 2004). However, it has not yet been reported whether the RcsC sensor is involved in regulating *ysa* and *ysp* gene expression. It might also be interesting to investigate whether there is crosstalk between the similar YsrS and RcsC sensors and the YsrR and RcsB response regulators. Can YsrS phosphorylate RcsB and/or can RcsC (perhaps via RcsD) phosphorylate YsrR? Finally, do the YsrRS and Rcs systems activate *ysa-ysp* gene expression only in response to identical signals, or might one or both systems also be able to induce in response to a signal that the other cannot?

### The Cpx system affects expression of the genes encoding the Ysc-Yop T3SS in *Y. pseudotuberculosis*

**Figure 2:**
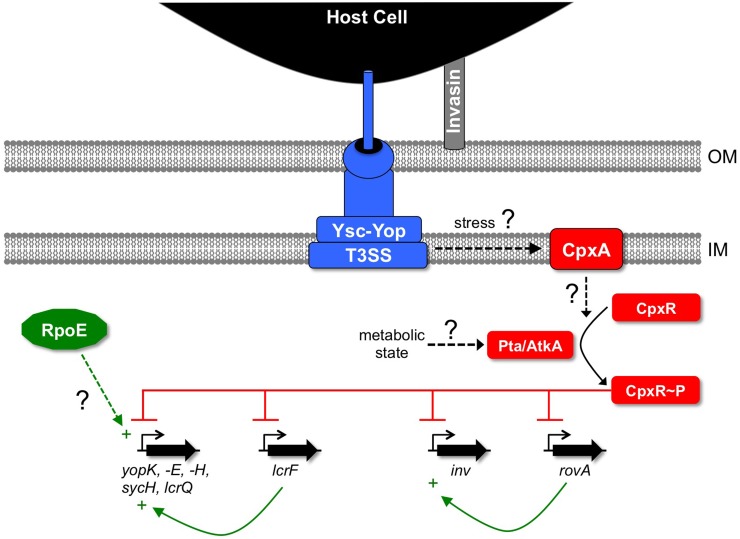
**Links between ESRs and regulation of the Ysc-Yop T3SS in *Y. pseudotuberculosis*.** CpxR~P down-regulates genes encoding components of the Ysc-Yop system by binding directly to their promoters. It also inhibits expression of the gene encoding the master positive regulator of the *ysc-yop* genes, *lcrF*, by the same mechanism. CpxR~P also represses expression of the genes encoding invasin (*inv*) and its positive regulator RovA. This further inhibits the function of the Ysc-Yop system by compromising the attachment to host cells that is required for efficient Yop delivery. It is not yet known what drives the phosphorylation of CpxR in this situation. Some evidence supports a role for elevated acetyl phosphate generated by the phosphotransacetylase (Pta)—acetate kinase (AtkA) pathway under as yet unknown metabolic conditions. It is also possible that envelope stress caused by assembly of the Ysc-Yop T3SS is sensed by CpxA, which then phosphorylates CpxR. RpoE has been implicated in positively regulating the Ysc-Yop T3SS although the mechanism remains unexplored. OM, outer membrane; IM, inner (cytoplasmic) membrane.

The next link between an ESR and the regulation of genes encoding a *Yersinia* T3SS was established for the Cpx and Ysc-Yop systems of *Y*. *pseudotuberculosis* (Figure 2). The core of the Cpx system is a classic two-component regulator pair consisting of the membrane bound sensor kinase CpxA and the cytoplasmic response regulator CpxR (for recent reviews see Hunke et al., 2012; Vogt and Raivio, 2012). Conditions that activate the Cpx response include alkaline pH, alterations of the cell envelope and the accumulation of misfolded proteins in the periplasm. This elevates the level of phosphorylated CpxR (CpxR~P), which activates the promoters of genes encoding envelope protein folding and degradation factors, in addition to various other functions. The Cpx system has been implicated in regulating virulence factors including two pili in *E. coli* (Hung et al., 2001; Hernday et al., 2004; Nevesinjac and Raivio, 2005), type IV secretion in *Legionella pneumophila* (Gal-Mor and Segal, 2003) and T3SSs in *Shigella sonnei* (Nakayama and Watanabe, 1995; Mitobe et al., 2005) and *E. coli* (Macritchie et al., 2012).

A global survey of the impact of the BaeSR, RpoE, and CpxAR ESRs on the *Y. pseudotuberculosis* Ysc-Yop T3SS revealed that inactivation of the BaeSR system has no effect (Carlsson et al., 2007a). In contrast, inactivation of RpoE does have an effect, but one that is difficult to interpret (discussed later). However, inactivating CpxA reduces Yop secretion and this has prompted investigation of the underlying mechanism. One clue is that CpxA can phosphorylate or dephosphorylate CpxR (reviewed in Hunke et al., 2012). The absence of CpxA in *E. coli* makes CpxR~P accumulate in non-inducing conditions due to phosphorylation by low molecular-weight phosphodonors (Danese et al., 1995). Therefore, the likely explanation for the effect of a *cpxA* null mutation on the Ysc-Yop T3SS is that CpxR~P accumulates, which then has an inhibitory effect. Indeed, a direct link has now been uncovered between the accumulation of CpxR~P and the down-regulation of genes encoding the Ysc-Yop T3SS (see below).

One reason Yop secretion is reduced in the *cpxA* null mutant is that some Ysc-Yop T3SS structural proteins are decreased (Carlsson et al., 2007a). However, the effect is not universal. Needle-associated components YscF and LcrV are affected, whereas proposed core components YscU and YscP are not. This is interesting because T3SS formation has been proposed to proceed in an ordered manner with core components assembling before the needle (e.g., Kimbrough and Miller, 2000; Sukhan et al., 2001). Therefore, it has been speculated that the Cpx system acts only beyond the integration of core components into the growing structure. In other words, there might be a Cpx-dependent assembly checkpoint. This is an enticing idea, but an observation that does not fit is that the *cpxA* null mutation reduces the amount of YscJ (Carlsson et al., 2007a). Recent work suggests that Ysc-Yop T3SS formation begins with localization of the YscC secretin component into the outer membrane followed by attachment of the cytoplasmic membrane rings composed of YscD and YscJ (Diepold et al., 2010). This makes YscJ one of the first components to assemble into the complex, well before the proposed Cpx-dependent checkpoint. Nevertheless, the YscJ level is reduced by a *cpxA* null mutation.

Another reason Yop secretion is reduced in the *cpxA* null mutant is that transcript levels are decreased (Carlsson et al., 2007a). Once again the effect is not universal. *yopE, -H, -K*, and *-D* transcripts are reduced whereas others, including the *ysc* structural genes, are not. This is similar to the phenotype caused by a feedback inhibition mechanism that reduces *yop* expression when Yop export is prevented (Cornelis et al., 1998). Feedback is mediated by accumulation of the inhibitory T3SS substrate LcrQ inside the cell. However, deleting *lcrQ* does not restore *yop* gene expression in a *cpxA* null mutant, arguing against the involvement of feedback inhibition (Carlsson et al., 2007a). Surprisingly, the *lcrQ* null mutation does fully restore Yop protein synthesis (cell associated protein) and secretion (protein in the supernatant) in the *cpxA* null mutant to wild type levels. The explanation for this is unclear, although even in a wild type strain an *lcrQ* null mutation increases Yop synthesis and secretion, even under non-permissive conditions (high Ca^2+^ concentration).

At least part of the mechanism underlying these *cpxA* null mutant phenomena relies on the accumulation of CpxR~P, the formation of which has been linked with low molecular-weight phosphodonors (Figure 2; Carlsson et al., 2007a,b; Liu et al., 2011, 2012). CpxR~P can bind directly to the promoters of some Ysc-Yop system-encoding genes to presumably reduce their activity (Liu et al., 2012). Interestingly, CpxR~P binds with relatively high affinity to the *yopK* and *lcrF* promoters, with somewhat lower affinity to the *yopH, yopE, sycH*, and *lcrQ* promoters and not at all to the *yopN*, *lcrG*, *yscA*, and *yscN* promoters. This suggests that CpxR regulates genes encoding late components of the system (Yops) and the master regulator LcrF directly, but not those encoding early/structural components (Ysc proteins). Perhaps this is also suggestive of a CpxR-dependent checkpoint.

As mentioned above, removing LcrQ from a *cpxA* null strain fully restores Yop secretion into the culture supernatant. However, it does not restore Yop-dependent cytotoxicity toward mammalian cells (Carlsson et al., 2007a). This is due to a phenomenon that provides another mechanism for CpxR to inhibit Ysc-Yop system effectiveness *in vivo*. A *cpxA* null mutation reduces attachment to host cells (Carlsson et al., 2007b), which is required for effective T3SS-dependent Yop delivery (e.g., Pettersson et al., 1996). This is mediated, at least in part, by CpxR~P binding to and inhibiting the promoter of *inv*, encoding the attachment factor invasin, as well as the *rovA* promoter, which encodes a positive regulator of *inv* expression (Liu et al., 2011). Thus, CpxR can control multiple virulence factors and interfere with Ysc-Yop system function directly and indirectly (Figure 2).

CpxR~P can directly control genes encoding the Ysc-Yop T3SS and attachment/invasion factors, as well as genes encoding positive regulators of each (LcrF and RovA, respectively). However, these effects occur in an artificial situation where the CpxA protein has been removed, leading to hyper-phosphorylation of CpxR in conditions where the Cpx ESR is not normally active. This raises the question of the physiological significance of these Cpx-dependent phenomena. In other words, can the native Cpx system have these inhibitory effects on virulence gene expression and if so, when? There is evidence to suggest that CpxR~P might influence some virulence factors in situations less artificial than the complete absence of CpxA. First, in a CpxA^+^ cell overproduction of the Cpx pathway-inducer NlpE down-regulates Inv and RovA protein levels, as well as Yop synthesis and secretion, in a CpxR-dependent manner (Liu et al., 2011, 2012). Second, although a *cpxR* null mutation in a CpxA^+^ cell does not affect Yop synthesis or secretion, it does enhance attachment of *Y. pseudotuberculosis* to HeLa cells, as well as cytotoxicity toward them (Carlsson et al., 2007b). This indicates that in a CpxA^+^ cell, endogenous CpxR is having a negative effect, at least on attachment factors. Nevertheless, the question of when endogenous CpxR exerts a negative effect on the Ysc-Yop system remains. Perhaps growth conditions affecting central metabolism play a role. Accumulation of CpxR~P in a *cpxA* null strain depends on the phosphotransacetylase (Pta)—acetate kinase (AtkA) pathway, from which the small molecular weight phospho-donor acetyl phosphate is derived (Liu et al., 2012). Therefore, there might be a physiologically relevant condition where acetyl-phosphate increases the CpxR~P concentration to a level where it can repress the *ysc-yop* genes (Figure 2).

Why would CpxR~P down-regulate the Ysc-Yop T3SS? One rationale is that it serves to keep Ysc-Yop production below a threshold that might compromise the cell envelope. However, some observations argue against this. First, a *cpxR* null mutation has not been reported to cause a growth/survival defect when the Ysc-Yop T3SS is active, as might be expected if down-regulation by CpxR~P reduces stress. However, conditions where endogenous CpxR represses the Ysc-Yop system would need to be found and used to compare *cpxR*^+^ and *cpxR* null strains. Second, why down-regulate genes encoding the Yops but not the Ysc structural proteins, which seem at least as likely to cause envelope stress? Third, acetyl phosphate could be the phospho-donor rather than CpxA (Liu et al., 2012). In that scenario CpxR-dependent down-regulation of the Ysc-Yop T3SS might be in response to a metabolic condition rather than envelope stress. This would perhaps fit with preferential down-regulation of the Yops because their production has been linked to metabolic activity in other studies (e.g., Schmid et al., 2009).

### Does the RPoE ESR regulate the Ysc-Yop T3SS?

RpoE (σ^E^) is a member of the ECF sigma factor family that is widely conserved in both Gram-negative and Gram-positive bacteria and often involved in responding to cell envelope stress (Helmann, 2002). In many cases activation of these systems involves a proteolytic cascade that releases the ECF sigma factor from an inhibitory interaction with a membrane associated anti-sigma factor (Brooks and Buchanan, 2008; Ho and Ellermeier, 2012). The RpoE system has been studied extensively, especially in *E. coli*. Inducing conditions include heat shock, oxidative and osmotic stress, and the overproduction and/or mislocalization of some outer membrane proteins. Numerous RpoE-induced genes have been identified in *E. coli* and many of them encode functions associated with mitigating envelope stress (e.g., Rhodius et al., 2006). RpoE has also been associated with the virulence properties of some pathogens (reviewed by Rowley et al., 2006). Finally, *rpoE* is an essential gene in some bacteria, including *E. coli* and *Y. enterocolitica* (De Las Penas et al., 1997a; Heusipp et al., 2003; Seo et al., 2007).

Some observations suggested that *rpoE* might also be essential in *Y. pseudotuberculosis* (Carlsson et al., 2007a). Therefore, Carlsson et al. investigated the effect of hyper-inducing RpoE by deleting the gene encoding the RpoE anti-sigma factor, RseA (De Las Penas et al., 1997b; Missiakas et al., 1997). The Δ*rseA* mutant had elevated Yop secretion, suggesting that RpoE might positively influence the Ysc-Yop T3SS (Figure 2). However, the problems associated with studying a potentially essential system mean that there has not yet been any mechanistic investigation into this phenomenon. The pleotropic effects of RpoE on cell envelope functions, as well as probable cross-regulation between the RpoE and Cpx ESRs, permit many hypotheses to be considered beyond direct regulation of *ysc-yop* promoters by RpoE, for which there is no evidence.

## An extracytolasmic stress response that is essential during production of the Ysc-Yop T3SS

**Figure 3:**
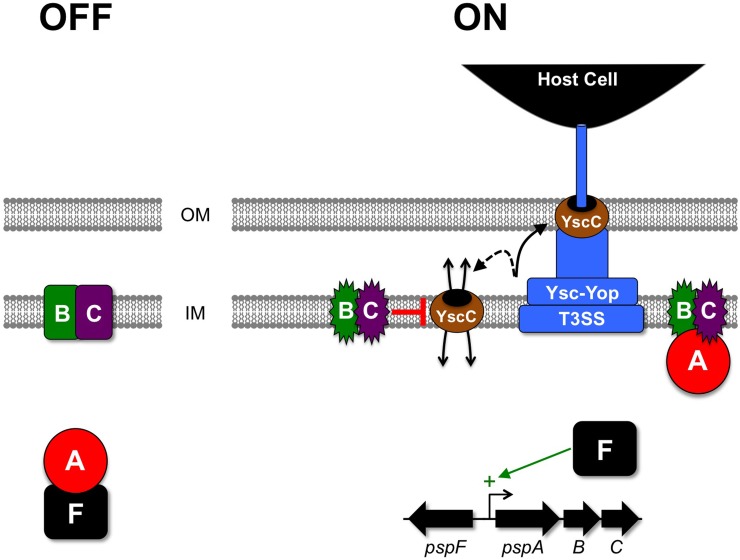
**Activation of the Psp ESR and relief of YscC-induced stress in *Y. enterocolitica*.** In the absence of Ysc-Yop T3SS production, PspA binds to the transcription factor PspF in the cytoplasm and inhibits it. When the Ysc-Yop apparatus is produced some of its secretin component (YscC) mislocalizes into the cytoplasmic membrane. This event is sensed by the PspBC proteins, which then switch into their active state and sequester PspA away from PspF, allowing it to activate the *pspA* operon promoter. Elevated levels of PspBC then stop mislocalized YscC from causing lethal cytoplasmic membrane permeability by an unknown mechanism. OM, outer membrane; IM, inner (cytoplasmic) membrane.

There are obvious rationales for regulation of T3SS production by an ESR, as discussed above. In particular, down-regulation by an ESR is the most obvious way to reduce T3SS-induced envelope stress. However, ESRs are known to have other ways of mitigating envelope stress besides reducing the synthesis of the stress-inducing protein(s). An example is the induction of cell envelope proteases and protein folding factors by the RpoE and Cpx systems. Apparently, the most extensively studied link between a T3SS and an ESR in *Yersinia* provides an instance where an ESR does not regulate T3SS production or function, but instead plays a critical role in alleviating/preventing T3SS-induced stress. This is the link between the Ysc-Yop T3SS and the Psp ESR of *Y. enterocolitica* (Figure 3).

The Psp system was discovered when it was found that a 25 kDa *E. coli* protein was highly produced during filamentous phage f1 infection (Brissette et al., 1990). The protein was named PspA (phage shock protein A) and it is encoded by the first gene of the *pspABCDE* operon (Brissette et al., 1991). Subsequent work has characterized the *E. coli* Psp system as a probable response to stress affecting the cytoplasmic membrane permeability barrier (reviewed by Model et al., 1997; Joly et al., 2010). It is induced by environmental shocks including heat, high osmolarity and organic solvents, and also by the production and mislocalization of some envelope proteins. Although the Psp ESR is not organized as a classic two-component system, it is made up of membrane and cytoplasmic proteins that form a signal transduction system for its activation (Figure 3). The Psp response has been implicated in *E. coli* biofilm formation, macrophage infection by *Shigella flexneri* and in *Salmonella enterica* serovar Typhimurium virulence (Darwin and Miller, 2001; Eriksson et al., 2003; Beloin et al., 2004; Lucchini et al., 2005; Karlinsey et al., 2010). This is in addition to its well-studied role in *Y. enterocolitica*, which is described below.

### The first description of the link between the *Y. enterocolitica* Psp and Ysc-Yop systems

A genetic screen to identify *Y. enterocolitica* virulence factors led to the isolation of a transposon-insertion mutant with an inability to survive in a mouse model of systemic infection (Darwin and Miller, 1999). This virulence defect is similar to that of a strain with a non-functional Ysc-Yop T3SS, which means that it renders *Y. enterocolitica* essentially avirulent. The mutant had a transposon insertion in the orthologue of the *E. coli pspC* gene, within the *pspA* operon. However, the *pspA* operons of both species are not identical (*pspABCDE* in *E. coli* and *pspABCD-ycjXF* in *Y. enterocolitica*). Early experiments to characterize the *Y. enterocolitica pspC* null mutant revealed a relationship between the Psp system and the Ysc-Yop T3SS, which offered an explanation for the impact on virulence (Darwin and Miller, 2001). Briefly, the *pspC* null mutation inhibits growth when the Ysc-Yop T3SS is produced (Darwin and Miller, 2001). More specifically, production and mislocalization of the YscC outer membrane component of the Ysc-Yop system is toxic to the *pspC* null mutant. This leads to a model where Ysc-Yop T3SS production during infection involves some inherent mislocalization of the endogenous YscC protein (Figure 3). This potentially toxic event is dealt with by the Psp system, but in a *psp* null mutant YscC mislocalization is lethal. However, there is no evidence to suggest that the Psp system has a regulatory effect on the Ysc-Yop system. For example, low Ca^2+^, 37°C-induced Yop secretion into culture medium is indistinguishable between wild type and *pspC* null strains (Darwin and Miller, 1999).

### The core components of the Psp system

The genes encoding the *Y. enterocolitica* Psp system are the *pspABCD-ycjXF* operon, the immediately adjacent *pspF*, and the unlinked *pspG* gene (Darwin and Miller, 2001; Green and Darwin, 2004). Non-polar deletions have associated only PspF, PspA, PspB, and PspC with robust phenotypes and so they have been considered the core components of the system (Darwin, 2007). In fact, all four of these proteins are involved in regulating the response (Figure 3), and PspA, -B and -C also have apparent roles in preventing and/or ameliorating envelope stress (recently reviewed by Yamaguchi and Darwin, 2012). The best-understood protein is PspF, which is a DNA-binding transcriptional regulator that activates the σ^54^-dependent promoters up-stream of *pspA* and *pspG* (Jovanovic et al., 1996; Green and Darwin, 2004). PspF is negatively regulated by PspA, which is thought to form an inhibitory complex with PspF in the cytoplasm (Yamaguchi et al., 2010). PspB and PspC are integral cytoplasmic membrane proteins that interact and are required for stress-dependent induction of the Psp response (Maxson and Darwin, 2006; Gueguen et al., 2009, 2011). The current model for activation is that during non-inducing conditions the Psp proteins are present at their basal level, with PspA inhibiting PspF in the cytoplasm and PspB and/or PspC serving as stress-sensors in the membrane (Figure 3). In response to an activating cue PspB and/or PspC sequester PspA to the membrane (Yamaguchi et al., 2010), which frees PspF to activate the *pspA* and *pspG* promoters. In contrast to the Cpx and RpoE systems that have many responsive genes, a remarkable feature of the Psp response is its extremely restricted transcriptional output. Transcriptional microarray analyses in three bacterial species have revealed that increased expression of the *pspA* operon and *pspG* might be the only direct consequence of increased PspF activity (Lloyd et al., 2004; Seo et al., 2007). The result is elevated levels of all the Psp proteins (except PspF), which is presumably important for stress relief. In particular, elevated concentrations of PspA, -B and -C are thought to be a critical feature of the response, although their individual physiological roles in stress relief probably have significant differences (e.g., Kleerebezem et al., 1996; Karlinsey et al., 2010; Horstman and Darwin, 2012).

### Secretin mislocalization kills *psp* null cells by disrupting the cytoplasmic membrane permeability barrier

As mentioned above, it is mislocalization of the YscC outer membrane component of the Ysc-Yop T3SS that is toxic to a *Y. enterocolitica pspC* null mutant (Figure 3). For example, the toxicity caused by production of YscC is exacerbated in the absence of its so-called pilot protein YscW, which is a situation that increases the mislocalization of YscC to the inner membrane (Darwin and Miller, 2001; Burghout et al., 2004). YscC is a member of a family known as secretins, which are multimeric pore forming outer membrane proteins found in various Gram-negative bacterial export systems (Genin and Boucher, 1994; Korotkov et al., 2011). A link between secretins and the Psp system actually dates back to its discovery in *E. coli*, because a single phage-encoded protein known as pIV is responsible for the induction of PspA synthesis during filamentous phage f1 infection (Brissette et al., 1990). pIV is a secretin used by the phage to export new viral particles across the outer membrane without causing cell lysis. However, pIV is particularly prone to mislocalization in the cell envelope (Russel and Kazmierczak, 1993; Daefler et al., 1997).

Although a link between secretins and induction of the Psp response was first described in *E. coli*, it was not until characterization of the Psp system in *Y. enterocolitica* that the toxicity of secretins to *psp* null strains was discovered (Darwin and Miller, 2001). Nevertheless, we now know that this secretin-toxicity also occurs in *E. coli* and *S*. Typhimurium *psp* null strains (Seo et al., 2007, 2009). However, a mechanism to explain how secretins kill *psp* null strains rapidly had not been described until recent work with *Y. enterocolitica* (Horstman and Darwin, 2012).

An assembled secretin multimer can insert into either membrane in *E. coli* and its mislocalization into the inner membrane collapses the membrane potential if the *pspA* operon is disrupted (Guilvout et al., 2006). Similarly, production of the YscC or YsaC secretins reduces the membrane potential in a *Y. enterocolitica psp* null strain (Horstman and Darwin, 2012). However, a reduced membrane potential might not be sufficient to explain the rapid cell death (Horstman and Darwin, 2012). This raised the possibility that a mislocalized secretin might cause a more profound effect on the cell envelope than permeability to protons or other small ions. Indeed, YscC production in a *Y. enterocolitica psp* null strain makes the cytoplasmic membrane permeable to molecules at least as large as the ~300 Da ortho-nitrophenyl-β-galactosidase (ONPG; Horstman and Darwin, 2012). Furthermore, microscopic examination of *psp* null cells overproducing YscC suggests that severe cytoplasmic shrinkage occurs in some of them, which is consistent with a severely compromised permeability barrier (Horstman and Darwin, 2012). However, the cytoplasmic membrane permeability of the *psp* null strain is almost abolished when YscC is co-produced with its pilot protein YscW, which reduces YscC mislocalization (Horstman and Darwin, 2012). Taking all of this together suggests that a secretin kills a *psp* null cell rapidly by mislocalizing to the cytoplasmic membrane and causing profound permeability. Finally, overproduction of a secretin that is prevented from multimerizing does not kill a *Y. enterocolitica psp* null strain (Horstman and Darwin, 2012). This raises the possibility that cytoplasmic membrane permeability results from leakage through the pore at the center of a secretin multimer.

### PspB and PspC are the proteins responsible for preventing secretin-induced toxicity in *Y. enterocolitica*

PspA is considered to be the master “effector” of the Psp response that mitigates the potential negative effects of an inducing stress. Several pieces of evidence support this in *E. coli*. For example, PspA is one of the most abundant cellular proteins when the system is activated by continuous production of the pIV secretin (Brissette et al., 1990). Furthermore, PspA has been associated with maintenance of the proton motive force *in vivo* (Kleerebezem et al., 1996) and with preventing leakage of protons from damaged membrane vesicles *in vitro* (Kobayashi et al., 2007). Despite all of this convincing evidence for an important physiological role for PspA, in *Y. enterocolitica* the loss of PspA does not cause sensitivity to secretin production (Darwin and Miller, 2001; Horstman and Darwin, 2012). Therefore, PspA is not required to combat the toxic effects of secretin mislocalization, at least in *Y. enterocolitica*.

Which core components of the Psp system do prevent secretin-toxicity? The answer has come from a number of studies, which have all led to the identification of the small integral cytoplasmic membrane proteins PspB and PspC as the critical factors (Maxson and Darwin, 2006; Gueguen et al., 2009; Horstman and Darwin, 2012). Thus, PspB and -C are dual function proteins required for both stress-responsive induction of *psp* gene expression (see above) and also for the physiological response to secretin-stress (Figure 3). In fact, in the case of PspC amino acid substitutions have been able to separate these two functions genetically (Gueguen et al., 2009). It is remarkable that these two small proteins can prevent all of the dramatic phenotypes associated with secretin production in a complete *psp* null strain. Of course, the obvious question is how they do it, but at least for now there is no answer. Perhaps the simplest hypothesis is that PspBC prevent a secretin from mislocalizing into the inner membrane. To date there is no evidence to support this, although it cannot yet be ruled out (Horstman and Darwin, 2012).

### What about PspA?

As mentioned above, a *pspA* null mutation does not render *Y. enterocolitica* sensitive to secretin mislocalization (Darwin and Miller, 2001). How can this be reconciled with the observation that PspA is the most abundant Psp protein when the system is induced and with the contention that it is an important physiological effector? Perhaps PspA is involved in counteracting relatively mild defects in cytoplasmic membrane permeability that might degrade ion gradients, whereas PspBC counteract much more severe damage such as the profound leakiness caused by a mislocalized secretin. Alternatively, PspA and PspBC might counteract different membrane defects, rather than different severities of the same defect. Regardless, PspA has been convincingly linked with maintenance of the PMF *in vivo* (Kleerebezem et al., 1996). In fact, in the intracellular pathogen *S*. Typhimurium this function of PspA is apparently essential for virulence. In this case, PMF maintenance by PspA appears to be critical to provide the energy that drives bacterial metal ion importers. These importers help the pathogen to acquire critical ions in the face of the host's natural resistance-associated macrophage protein 1 (Nramp-1) that seeks to deplete them from the *Salmonella*-containing vacuole (Karlinsey et al., 2010).

### Some pressing questions about the *Y. enterocolitica* Psp response

An obvious question, touched on above, is how PspB and PspC prevent secretin-induced bacterial cell death. The simple hypothesis of preventing secretins from mislocalizing is not supported by current data, but also not yet disproven. Of course, other more complex possibilities can be considered. For example, PspB and PspC could disrupt secretin multimers to prevent toxicity, because it is known that a secretin that cannot multimerize is not toxic to a *psp* null strain (Horstman and Darwin, 2012). However, once again there is no current data to support that. Another possibility is suggested by recent experiments that revealed FtsH-dependent degradation of PspC when PspB is absent (Singh and Darwin, 2011). This is interesting because PspC production is toxic in the absence of its binding partner PspB. Therefore, FtsH dependent-degradation might have evolved as a quality control mechanism to counteract the potential for PspC toxicity. This situation is similar to the FtsH dependent-degradation of the cytoplasmic membrane proteins SecY and AtpB in *E. coli* when they cannot form a complex with their normal binding partners (Kihara et al., 1995; Akiyama et al., 1996). SecY and AtpB are components of complexes that transport proteins or protons, respectively, across the cytoplasmic membrane. In isolation, their transport functions might disrupt membrane permeability, raising the need for destruction of the uncomplexed proteins by FtsH. By analogy, PspC might also have a membrane-permeabilization and/or transport function that is normally tightly regulated by PspB, but becomes deleterious when PspB is absent. This hypothetical function of PspC might be important for mitigating secretin-toxicity. However, all of this remains highly speculative with current data.

Another interesting question is the nature of the signal that triggers increased *psp* gene expression. It used to be thought that a decreased PMF might be the inducing signal. However, this now appears unlikely (Engl et al., 2011; Horstman and Darwin, 2012). Clearly, secretin mislocalization disrupts the cytoplasmic membrane permeability barrier in a *psp* null strain. Therefore, the Psp response might be activated by increased cytoplasmic membrane permeability. However, some observations argue against this. First, secretin overproduction induces *psp* gene expression in a wild type *psp*^+^ cell but does not render the cytoplasmic membrane permeable to ONPG or decrease the membrane potential. Second, some non-secretin proteins are potent inducers of the Psp response (Maxson and Darwin, 2004) but they do not affect membrane potential or permeability in either *psp*^+^ or even *psp* null cells (Horstman and Darwin, 2012). Third, secretin overproduction activates *psp* gene expression but does not activate the expression of any other genes (Lloyd et al., 2004; Seo et al., 2007). This suggests that the inducing trigger is highly specific, which does not fit well with something as potentially pleotropic as general membrane permeability.

## Concluding remarks

Recent work with various pathogenic bacteria has begun to uncover connections between T3SS and ESRs that include effects of these stress responses on the expression and function of T3SSs, and on mitigating the stress they can cause. Work in *Yersinia* has provided examples of all of these: positive regulation of genes encoding the Ysa-Ysp system by at least one component of the Rcs ESR in *Y. enterocolitica*; negative regulation of the Ysc-Yop system by CpxR in *Y. pseudotuberculosis*; and relief of Ysc-Yop system-induced envelope stress by the Psp ESR in *Y. enterocolitica*. In addition, RpoE might also control the level of the Ysc-Yop system in *Y. pseudotuberculosis*, although in this case the mechanism remains completely unexplored.

Challenges and questions for the future remain. Indeed, research into the relationship between ESRs and T3SSs in *Yersinia* is still very much in its infancy, essentially encompassing only approximately the last decade. Obviously, these questions include some of the specific points touched on above such as the details of the regulatory cascade linking RcsB and the Ysa-Ysp T3SS, identifying the conditions where endogenous CpxR down-regulates the Ysc-Yop system, understanding the mechanism by which RpoE impacts the Ysc-Yop system, and discovering exactly how PspB and PspC mitigate YscC-induced stress. More general areas are also worthy of investigation. Notably, one potential complication is that much of the work has been in different *Yersinia* species and has also examined the impact of one ESR on only one export system. It is possible that these ESR systems do not act identically in the different *Yersinia*. In fact, there is a suggestion that the role of the Cpx system in *Y. enterocolitica* might have some physiological distinctions from that in *Y. pseudotuberculosis* (Heusipp et al., 2004; Ronnebaumer et al., 2009). In addition, effects of one ESR might extend to multiple export systems, especially in the case of the Cpx and RpoE systems. For example, does CpxR down-regulate the Ysa-Ysp T3SS in *Y. enterocolitica*, and/or other secretion systems such as type 2 exporters and type 4 pili? Another important area is coordination and crosstalk between the different ESRs that must almost certainly function simultaneously, especially during a host infection when it seems likely that many are active (e.g., Darwin and Miller, 2001; Heusipp et al., 2003; Carlsson et al., 2007a,b).

ESRs might not be considered so-called classic virulence factors because they are found in both pathogens and non-pathogens alike. However, it is becoming more and more clear that they are intimately linked to the function of systems that are used during host infection specifically, and to ensuring bacterial survival in this environment. Understanding exactly how they connect with critical virulence factors such as the T3SS has clear significance and holds the promise of designing interventions that might disrupt these connections. As for many other aspects of bacterial pathogenesis, *Yersinia* is proving to be an excellent model to investigate these very important questions.

### Conflict of interest statement

The authors declare that the research was conducted in the absence of any commercial or financial relationships that could be construed as a potential conflict of interest.
